# Basal Vascular Smooth Muscle Cell Tone in eNOS Knockout Mice Can Be Reversed by Cyclic Stretch and Is Independent of Age

**DOI:** 10.3389/fphys.2022.882527

**Published:** 2022-04-28

**Authors:** Sofie De Moudt, Jhana O. Hendrickx, Guido R. Y. De Meyer, Wim Martinet, Paul Fransen

**Affiliations:** Laboratory of Physiopharmacology, University of Antwerp, Antwerp, Belgium

**Keywords:** cyclic stretch, vascular smooth muscle, calcium, arterial stiffness, endothelial nitric oxide synthase

## Abstract

**Introduction and Aims:** Endothelial nitric oxide synthase (eNOS) knockout mice develop pronounced cardiovascular disease. In the present study, we describe the alterations in aortic physiology and biomechanics of eNOS knockout and C57Bl/6 control mice at 2–12 months of age, including a thorough physiological investigation of age and cyclic stretch-dependent VSMC contractility and aortic stiffness.

**Methods and Results:** Peripheral blood pressure and aortic pulse wave velocity were measured *in vivo*, and aortic biomechanical studies and isometric contractions were investigated *ex vivo*. Age-dependent progression of aortic stiffness, peripheral hypertension, and aortic contractility in eNOS knockout mice was absent, attenuated, or similar to C57Bl/6 control mice. Voltage-gated calcium channel (VGCC)-dependent calcium influx inversely affected isometric contraction and aortic stiffening by α_1_-adrenergic stimulation in eNOS knockout mice. Baseline aortic stiffness was selectively reduced in eNOS knockout mice after *ex vivo* cyclic stretch exposure in an amplitude-dependent manner, which prompted us to investigate cyclic stretch dependent regulation of aortic contractility and stiffness. Aortic stiffness, both in baseline conditions and after activation of vascular smooth muscle cell (VSMC) contraction, was reduced with increasing cyclic stretch amplitude. This cyclic stretch dependency was attenuated with age, although aged eNOS knockout mice displayed better preservation of cyclic stretch-dependency compared to C57Bl/6 control mice. Store operated calcium entry-medicated aortic stiffening as induced by inhibiting sarcoplasmic reticulum calcium ATPase pumps with 10 µM CPA was most pronounced in the aorta of aged mice and at low cyclic stretch amplitude, but independent of eNOS. Basal aortic tonus and VSMC depolarization were highly dependent on eNOS, and were most pronounced at low cyclic stretch, with attenuation at increasing cyclic stretch amplitude.

**Conclusion:** eNOS knockout mice display attenuated progression of arterial disease as compared to C57Bl/6 control mice. Basal VSMC tone in eNOS knockout mice could be reduced by *ex vivo* exposure to cyclic stretch through stretch-dependent regulation of cytosolic calcium. Both baseline and active aortic stiffness were highly dependent on cyclic stretch regulation, which was more pronounced in young versus aged mice. Other mediators of VSMC contraction and calcium handling were dependent on cyclic stretch mechanotransduction, but independent of eNOS.

## Introduction

As the primary lining of every blood vessel in the body, the endothelium comprises a crucial interface between blood and tissue, and governs numerous vascular responses (e.g., contractile tone, leukocyte interaction, proliferation). Endothelial cells exert their function by secretion of small molecule, peptide, and protein mediators with autocrine and paracrine effects on neighboring cells (Segers et al., 2018). These factors include, among others, prostacyclins, angiotensin II, and endothelin I. The most studied molecule however is nitric oxide (NO), produced by enzymatic activity of endothelial nitric oxide synthase (eNOS), utilizing l-arginine as a substrate and generating NO from its terminal guanidine nitrogen (Atochin and Huang, 2010). NO was the first gaseous molecule ever accepted as a cell signaling mediator, and the discovery of its vasodilatory function was awarded the 1998 Nobel prize of physiology and medicine (SoRelle, 1998).

NO is produced in both basal and stimulated conditions (Leloup A. J. A. et al., 2015), and its importance is highlighted by an abundance in regulatory systems (Zhao et al., 2015). eNOS function is regulated on the transcriptional level (Fulton, 2016), by substrate and cofactor bioavailability (Siragusa and Fleming, 2016), post-translational modifications (e.g., S^1177^ phosphorylation) (Fulton, 2016), dimerization (Alderton et al., 2001), interactions with calcium and calcium-binding proteins (Busse and Mulsch, 1990), and subcellular localization (Bucci et al., 2000; Drab et al., 2001). Furthermore, eNOS is activated by a multitude of stimuli, such as shear stress, acetylcholine, bradykinin, and insulin (Zhao et al., 2015). Finally, endothelial dysfunction - often defined as impaired NO bioavailability - acts as a common player in most cardiovascular (CV) risk factors (Rajendran et al., 2013), underlining the importance of endothelial NO signaling in CV ageing and disease. Such risk factors include hypertension (Panza et al., 1990; Perticone et al., 2001), arterial stiffness (Leloup et al., 2019), smoking (Korneeva and Sirotin, 2017), diabetes (Calver et al., 1992), obesity (Engin, 2017), inflammation (Yang et al., 2016), and oxidative stress (Cai and Harrison, 2000).

As an absolute and genetic model of endothelial dysfunction, the eNOS knockout mouse was first described in 1995 (Huang et al., 1995) and has been widely used in cardiovascular research ever since. eNOS knockout mice are fertile and display a normal anatomy (Huang et al., 1995), although with reduced body weight (Razny et al., 2010). Their CV disease phenotype includes hypertension (Yang et al., 1999; Van Vliet et al., 2003), elevated pulse wave velocity (Leloup et al., 2014), increased platelet aggregation (Freedman et al., 1999), high sensitivity to ischemic stroke and atherosclerosis (Huang et al., 1996; Chen et al., 2001; Kuhlencordt et al., 2004), and greater neointima formation after vascular injury (Mooradian et al., 1995; Moroi et al., 1998). In the heart, eNOS is expressed in both endothelial cells and cardiomyocytes (Tirziu and Simons, 2008), and eNOS gene deletion results in a decreased heart rate (Bannister et al., 2011), left ventricular hypertrophy (Bannister et al., 2011), and impaired left ventricular relaxations (Vignon-Zellweger et al., 2011). Furthermore, eNOS knockout mice show fewer mitochondria, changes in thermogenesis and energy metabolism (Nisoli et al., 2003; Le Gouill et al., 2007), altered blood glucose, cholesterol, insulin, and leptin (Razny et al., 2010), renal dysfunction (Tsuprykov et al., 2015), and neurochemical abnormalities (Demas et al., 1999; Dere et al., 2002). Despite this wide array in phenotypic data, limited information is off yet available on the exact changes in aortic function which underlie the pronounced vascular phenotype in eNOS knockout mice.

Our research group previously published a biomechanical characterization of aortic alterations in eNOS knockout mice at 5 months of age (Leloup et al., 2020), showing reduced aortic diameter and increased isobaric aortic stiffness, heightened α_1_-adrenoreceptor-mediated contraction-dependent aortic stiffening, increased basal VSMC calcium load, and reduced contractions mediated through calcium release from intracellular stores. In the present study, we investigated whether this arterial eNOS phenotype is altered with aging by performing a longitudinal characterization of eNOS knockout mice from 2 to 12 months of age (compared to age-matched C57Bl/6 control groups). Additionally, since calcium signaling has been shown to be dependent on cyclic stretch mechanotransduction (Kudryavtseva et al., 2013; Gilbert et al., 2014; Misarkova et al., 2016; Ahmed and Warren, 2018) and VSMC calcium signaling is aberrant in the arteries of eNOS knockout mice (Leloup et al., 2020), the cyclic stretch-sensitivity of VSMC calcium signaling alterations in eNOS knockout mice was ascertained, both in young and aged mice to assess possible age-related effects.

## Materials and Methods

### Animals

All animal experiments were approved by the Ethical Committee of the University of Antwerp and were conducted in accordance to the Guide for the Care and Use of Laboratory Animals, published by the National Institutes of Health (NIH Publication No. 85-23; Revised, 1996). All mice were bred and housed in the animal facility of the University of Antwerp, with a 12 h/12 h light-dark cycle and had free access to water and standard chow. The following groups of animals were used in the present study: 1) **Experiment 1:** eNOS knockout mice of 2 (*n* = 10), 4 (*n* = 10), 6 (*n* = 21), 9 (*n* = 15), and 12 (*n* = 17) months of age and C57Bl/6 control mice of 2 (*n* = 14), 4 (*n* = 11), 6 (*n* = 25), 9 (*n* = 10), and 12 (*n* = 20) months of age, 2) **Experiment 2:** eNOS knockout (*n* = 3) and C57Bl/6 control (*n* = 3) mice of 5 months of age, and 3) **Experiment 3:** 4-month old eNOS knockout (*n* = 6, “young”), 4 month old C57Bl/6 control (*n* = 6, “young”), 13-month old eNOS knockout (*n* = 4, “aged”), and 14-month old C57Bl/6 control (*n* = 4, “aged”) mice. When mice were under deep anesthesia (pentobarbital sodium, 75 mg/kg ip; Sanofi, Belgium), mice were euthanized by perforation of the diaphragm. The thoracic aorta was carefully removed and stripped of adherent tissue. Next, aortic rings of 2 mm width were cut starting at the diaphragm for *ex vivo* aortic biomechanics and/or isometric reactivity studies. In Experiment 3, additional 2-mm thoracic aorta segments were snap-frozen for RNA isolation.

### *In Vivo* Blood Pressure and Pulse Wave Velocity

In **Experiment 1**, mice underwent cardiovascular tests 1 week before sacrifice. Peripheral blood pressure was measured with a CODA tail-cuff method as previously described (Fransen et al., 2016). In brief, a pressure-volume sensor was attached distally to an occluding cuff to the tail of conscious restrained mice for blood pressure recording. Systolic and diastolic blood pressure were measured on three consecutive days, of which the final measurement was used. Abdominal aorta pulse wave velocity (aPWV) was measured as described by Di Lascio et al. (2014). In short, B-mode images of aortic diameter and pulsed wave Doppler analysis of velocity were acquired and averaged over several cardiac cycles. aPWV was calculated as dV/2dln(D) (with dV, velocity change; and dln(D), the variation of the natural logarithm of diameter).

### Isometric Reactivity Studies of the *Ex Vivo* Thoracic Aorta

In **Experiment 1**, 2-mm aortic rings were mounted between two parallel wire hooks in a 10-ml organ bath containing Krebs-Ringer solution (composition (mM): NaCl 118; KCl 4.7; CaCl_2_ 2.5; KH_2_PO_4_ 1.2; MgSO_4_ 1.2; NaHCO_3_ 25; CaEDTA 0.025; glucose 11.1). The solution was continuously heated to 37°C and aerated with a 95% O_2_/5% CO_2_ gas mixture to maintain a pH of 7.4. A preload of 20 mN was applied to approximate normal physiological stretch at a mean blood pressure of 100 mmHg (De Moudt et al., 2017), and isometric contractions and relaxations were measured by means of a Statham UC2 force transducer (Gould, United States). Contractions were induced by concentration-response stimulation with α_1_-adrenergic agonist phenylephrine (PE, 3 nM to 10 µM), in the absence and presence of voltage-gated calcium channel (VGCC) agonist BAY-K8644 (30 nM). Subsequently, VGCC were blocked with 35 µM diltiazem to assess the contribution of VGCC to PE-induced contractions. Endothelium-dependent and –independent relaxations were induced by concentration-response stimulation with acetylcholine (ACh, 3 nM to 1 µM) and diethylamine NONOate (DEANO, 0.3 nM-10 µM), respectively, in PE-precontracted aortic rings. For DEANO-induced relaxations, 300 μM N-Ω-Nitro-l-arginine methyl ester hydrochloride (l-NAME, NOS blocker) was also added to the organ bath to exclude effects of endogenously produced NO. Basal NO levels were quantified as the relative difference in PE-induced contractile force in the absence and presence of l-NAME. Finally, to avoid extra cellular calcium influx, Krebs-Ringer solution was replaced by a solution lacking calcium (0Ca Krebs), and a transient IP_3_-mediated contraction was induced by 2 μM PE as previously described (Fransen et al., 2015). IP_3_-mediated contractions were studied in parallel in two aortic rings and averaged for the analysis. All concentration-response curves were fitted with a non-linear 4-parameter equation, to obtain values for maximal effect and half-maximal effective or inhibitory concentration (EC_50_ or IC_50_).

### *Ex Vivo* Isobaric Measurement of Aortic Stiffness

2-mm aortic rings were mounted in a Rodent Oscillatory Tension Set-up for Arterial Compliance (ROTSAC), between two parallel wire hooks in a 10-ml organ bath containing Krebs-Ringer solution. The upper wire hook was connected to a force-length transducer, and segments were continuously stretched between alternating preloads corresponding to the “systolic” and “diastolic” transmural pressures at a physiological frequency of 10 Hz to mimic the physiological heart rate in mice (600 bpm) (Leloup et al., 2016). At any given pressure, calibration of the upper hook allowed for the calculation of the diastolic and systolic vessel diameter (mm) and Peterson modulus (E_p_). E_p_ was defined as the pulse pressure divided by the relative diameter change (E_p_ = D_0_*ΔP/ΔD), and can be interpreted as the pressure change that is required to increase aortic diameter by 100%. Aortic stiffness was always assessed in isobaric conditions.

In the main aging study, **Experiment 1**, two aortic segments of each mouse were mounted in ROTSAC organ chambers, and aortic stiffness was measured at oscillating pressures of 60–100, 80–120, 100–140, and 120–160 mmHg. Contraction and relaxation of vessel segments were elicited as described above to assess different players in active contraction-dependent aortic stiffening.

In **Experiment 2**, four aortic segments of each mouse were mounted in ROTSAC organ chambers, and subjected to normal (calculated 80–120 mmHg) or high (calculated 80–150 mmHg) pulse pressure oscillations during 1 h, to investigate the effect of cyclic stretch exposure on isobaric aortic stiffness. Isobaric E_p_ was measured before and after the 1-h stretch exposure, and the effect of the cyclic stretch exposure was calculated as the relative difference in E_p_ at the start and end of the experiment. No vasoactive agents were used in this experiment.

In **Experiment 3**, two aortic segments of each mouse were mounted in ROTSAC organ chambers to investigate the cyclic stretch-dependent regulation of VSMC calcium signaling, by exposing aortic segments subsequently to low (calculated 80–90 mmHg), normal (calculated 80–120 mmHg), and high (calculated 80–150 mmHg) cyclic stretch amplitudes, in the presence of various vasoactive drugs. Each level of cyclic stretch was maintained for 5 min prior to measurement, and a 10-min normalization period at 80–120 mmHg was employed prior to addition of a new vasoactive agent. Vascular contraction was induced using α_1_-adrenergic agonist phenylephrine PE (2 µM), in the absence or presence of l-NAME (300 µM) to inhibit endothelial NO production. Calcium channel/pump activity was controlled by addition of VGCC agonist BAY-K8644 (30 nM), VGCC blocker diltiazem (35 µM), or sarcoplasmic reticulum calcium ATPase (SERCA) pump inhibitor cyclopiazonic acid (CPA, 10 µM). To remove all VSMC contractile tone, Krebs-Ringer solution was replaced by a solution lacking calcium (0Ca Krebs) for the final measurement. All measurements were performed after steady-state conditions were reached.

#### Quantitative Reverse Transcriptase PCR

In **Experiment 3**, aortic tissue was snap-frozen for qPCR analysis. RNA isolation and reverse transcription were carried out using a NucleoSpin^®^ RNA kit (Macherey-Nagel) and a SensiFAST™ cDNA Synthesis Kit (Bioline), respectively. The resulting cDNA was subjected to qPCR reactions using commercially available Taqman assays specific for STIM1 (ThermoFisher, Mm01158413_m1), Orai1 (ThermoFisher, Mm00774349_m1), ITPR3 (ThermoFisher, Mm01306070_m1), Cacna1C (ThermoFisher, Mm01188822_m1), ATP2A2 (ThermoFisher, Mm01201431_m1), ATP2B1 (ThermoFisher, Mm01245805_m1).

#### Statistical Analysis

Data was expressed as mean ± SEM, with *n* representing the number of biological replicates. All analyses were performed using GraphPad Prism (version 8, GraphPad Software) and a significance level of 5% was set to identify statistically significant changes. Statistical testing was performed using student *t*-test, one-way ANOVA, two-way ANOVA, and three-way ANOVA as indicated in the figure legends. A Tukey multiple testing correction was employed for post-hoc testing of the ANOVA tests. Age-dependent changes were statistically tested using simple linear regression analysis with F-testing to ascertain if the slope is significantly different from zero.

## Results

### Limited Age-Dependent Progression of Arterial Disease in eNOS Knockout Mice

**FIGURE 1:**
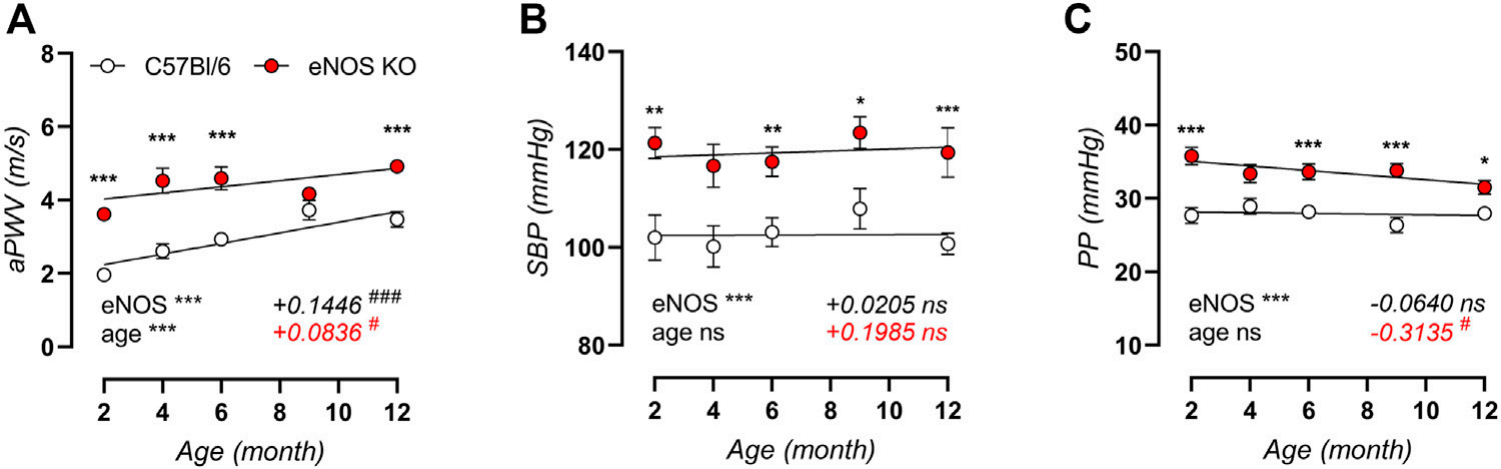
*In vivo* arterial aging is less pronounced in eNOS knockout versus C57Bl/6 control mice. Measurements of aPWV **(A)**, systolic blood pressure (SBP, **(B)**), and pulse pressure (PP, **(C)**) were performed in aging eNOS knockout (*n* > 10) and C57Bl/6 control (*n* > 9) mice. Age-dependent changes were analyzed using linear regression (slope displayed, bottom right). An F-test was used to assess if the slope was different from zero. Statistical analysis using two-way ANOVA. Overall significance (bottom left) and post-hoc significance (in graph) are listed. ANOVA: ns, *p* > 0.05; *, *p* < 0.05; **, *p* < 0.01; ***, *p* < 0.001. F-test: ns, *p* > 0.05; #, *p* < 0.05; ###, *p* < 0.001.

High-frequency ultrasound analysis of *in vivo* aortic stiffness (Figure 1A) revealed a significant, progressive increase in aPWV with age in control C57Bl/6 mice, with a slope of +0.1446 m/s per month. Although aortic stiffness was significantly increased in eNOS knockout mice compared to C57Bl/6 control mice, the age-dependent aPWV increase displayed a ∼40% lower slope for the aPWV-age relationship, indicating less pronounced age-dependent aortic stiffening in eNOS knockout mice. Measurement of peripheral blood pressure revealed significantly elevated systolic blood pressure in eNOS knockout mice (Figure 1B), but blood pressure did not increase with age in eNOS knockout or C57Bl/6 control mice from 2 to 12 months of age. Similarly, pulse pressure (Figure 1C) was increased in eNOS knockout mice, but did not increase with age in eNOS knockout or C57Bl/6 control mice. To the contrary, linear regression of pulse pressure in eNOS knockout mice revealed a slightly negative slope (−0.3135 mmHg per month) for the PP-age relationship in eNOS knockout mice, which was significantly different from zero. In C57Bl/6 control mice, the slope for the PP-age relationship was not significantly different from zero.

**FIGURE 2:**
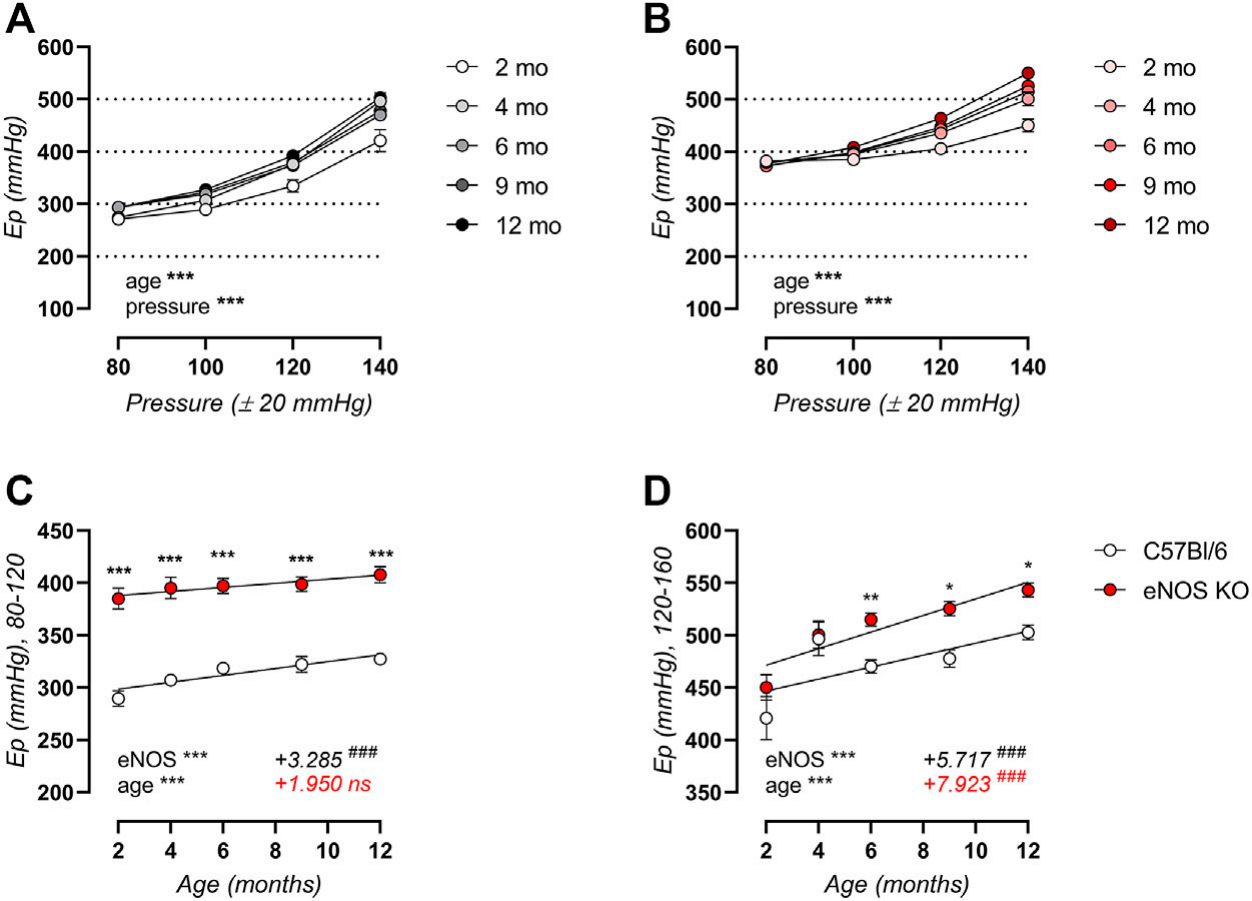
*Ex vivo* arterial aging is less pronounced in eNOS knockout versus C57Bl/6 control mice at physiological distending pressure. Peterson modulus (E_p_) in baseline Krebs-Ringer conditions over a broad pressure range is displayed for aging C57Bl/6 control (*n* > 9) **(A)** and eNOS knockout (*n* > 9) **(B)** mice. Detailed views of E_p_ at physiological (calculated 80–120 mmHg) **(C)** and high (calculated 120–160 mmHg) **(D)** distending pressure are shown. Age-dependent changes in **(C,D)** were analyzed using linear regression (slope displayed, bottom right). An F-test was used to assess if the slope was different from zero. Statistical analysis using two-way ANOVA. Overall significance (bottom left) and post-hoc significance (in graph) are listed. No post-hoc significance is listed in **(A,B)**. ANOVA: ns, *p* > 0.05; *, *p* < 0.05; **, *p* < 0.01; ***, *p* < 0.001. F-test: ns, *p* > 0.05; ###, *p* < 0.001.

Aortic stiffness was further investigated in *ex vivo* ROTSAC organ chambers, revealing significant age-related aortic stiffening in both C57Bl/6 control (Figure 2A) and eNOS knockout (Figure 2B) mice over a broad pressure range, even though arterial aging in eNOS knockout mice seemed less pronounced at low to physiological pressure. This is illustrated in Figures 2C,D, showing that aortic stiffness was increased in eNOS knockout mice at both calculated 80–120 mmHg and 120–160 mmHg distending pressure across all ages. However, age-dependent aortic stiffening at physiological pressure (Figure 2C) was less pronounced in eNOS knockout mice versus C57Bl/6 controls, as indicated by a reduced slope in the E_p_-age relationship. Age-dependent aortic stiffening in eNOS knockout mice at high distending pressure (Figure 2D) was slightly higher compared to C57Bl/6 mice.

**FIGURE 3:**
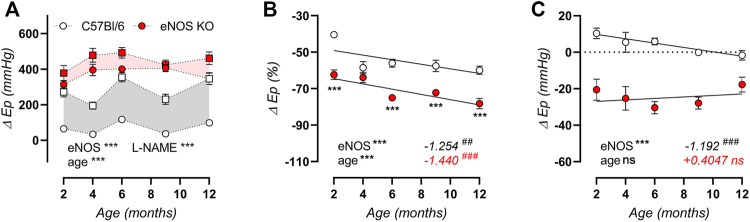
Active contraction-dependent aortic stiffening is not affected by age in eNOS knockout mice. The difference in isobaric aortic stiffness (E_p_) at calculated 80–120 mmHg distending pressure is shown for contraction induced with 2 μM PE in the absence (circles) and presence (squared) of 300 μM l-NAME to inhibit basal NO production **(A)**. Grey and light red shading indicate the amount of contraction-dependent stiffening that is suppressed by basal NO in C57Bl/6 mice and eNOS knockout control mice, respectively. The percentage inhibition of contraction-dependent stiffening at calculated 80–120 mmHg distending pressure by 35 µM diltiazem in 2 μM PE precontracted aortic rings (in the presence of 300 μM l-NAME) was used to indicate VGCC contribution **(B)**. The effect on isobaric stiffness at calculated 80–120 mmHg distending pressure by removal of all active VSMC tone by 10 µM DEANO compared to baseline Krebs-Ringer conditions was plotted **(C)**. Age-dependent changes in B and C were analyzed using linear regression (slope displayed, bottom right). An F-test was used to assess if the slope was different from zero. Statistical analysis using three-way ANOVA **(A)** or two-way ANOVA **(B,C)**. Overall significance (bottom left) and post-hoc significance (in graph) are listed. No post-hoc significance is listed in **(A)**. ANOVA: ns, *p* > 0.05; **, *p* < 0.01; ***, *p* < 0.001. F-test: ns, *p* > 0.05; ##, *p* < 0.01; ###, *p* < 0.001.

We previously demonstrated that 5-month old eNOS knockout mice displayed increased α_1_-adrenergic contraction-dependent stiffening in response to stimulation with 2 μM PE, increased baseline VSMC cytoplasmic calcium levels, and increased VGCC function (Leloup et al., 2020). Here, we could confirm these changes. Additionally, we could demonstrate that although these parameters change with age in C57Bl/6 control mice, this age-dependency was either absent or similar to C57Bl/6 controls in eNOS knockout mice. Figure 3A shows the contraction-dependent stiffening after stimulation with 2 μM PE, in the absence or presence of NOS-blocker l-NAME to inhibit basal NO production. As expected, inhibition of NO production had limited effects in eNOS knockout mice, whereas basal NO showed a great capacity of suppressing contraction-dependent stiffening in C57Bl/6 mouse aortic segments. In both conditions, active aortic stiffening was more pronounced in eNOS knockout versus C57Bl/6 control mice. Age-dependency was assessed using non-linear regression of the ΔE_p_-age relationship, demonstrating a non-significant trend toward increased contraction-dependent stiffness in C57Bl/6 mice in the absence (slope: +2.549 mmHg per month, F-test: *p* = 0.1954) and presence (slope: +7.085 mmHg per month, F-test: *p* = 0.0909) of NOS-blocker l-NAME. In contrast, in eNOS knockout mice, this analysis revealed a slope of +1.084 mmHg per month (F-test: *p* = 0.7557) and +3.209 mmHg per month (F-test: *p* = 0.4872), respectively, which were not significantly different from zero. In Figure 3B, the contribution of VGCC to the PE-induced contraction-dependent stiffening was investigated by inhibition of VGCC with 35 µM diltiazem (in the presence of NOS blocker l-NAME). A clear age-dependent increase in VGCC contribution was demonstrated, which was similar in eNOS knockout and C57Bl/6 control mice. In Figure 3C, all contractile tone was removed by administration of an excessive concentration of exogenous NO donor DEANO (10 µM). Compared to baseline Krebs-Ringer conditions, this induced a significant decrease in E_p_ in eNOS knockout mice, indicating a high basal contractile calcium content in the cytoplasm of VSMC. As shown in Figure 3C, no decrease in E_p_ upon DEANO administration was observed in C57Bl/6 control mice. The high basal calcium load in VSMC of eNOS knockout mice was not affected by age.

### Isometric Reactivity Analysis Confirms Impaired Endothelial NO Production in eNOS Knockout Mice

**FIGURE 4:**
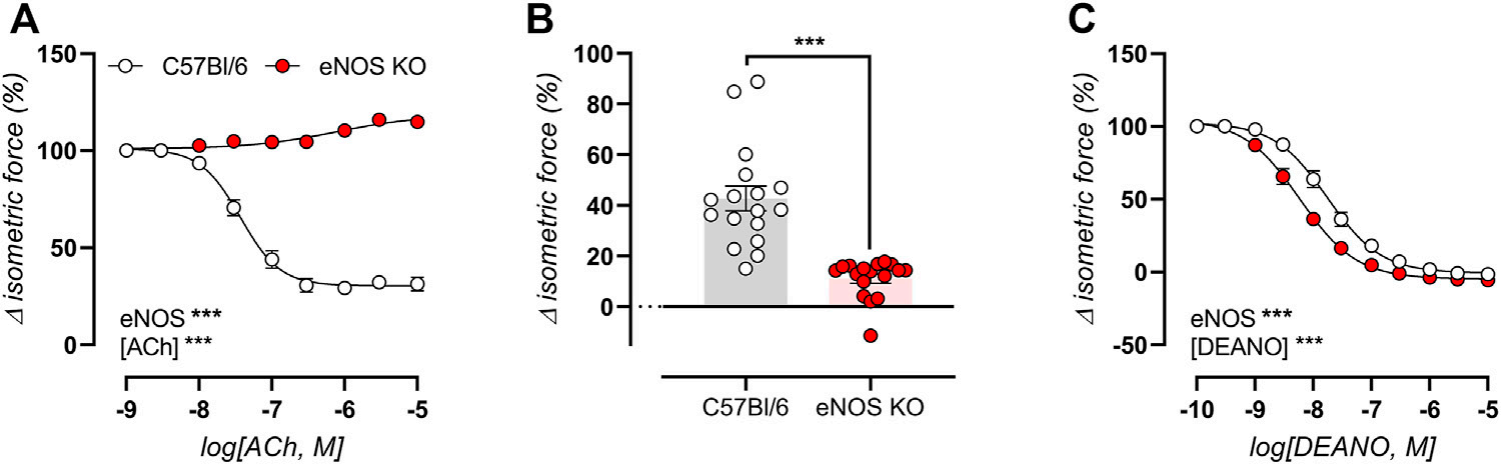
Impaired endothelial NO production in eNOS knockout mice. Endothelial function was assessed in 6-month old eNOS knockout (*n* = 17) and C57Bl/6 control (*n* = 17) mice, by concentration-response stimulation with ACh in 2 µM PE-preconstricted aortic rings **(A)**, and by calculating of basal NO production as the relative difference in 2 µM PE-induced contraction in the absence and presence of NO blocker l-NAME (300 µM) **(B)**. The sensitivity of VSMC to exogenous NO was assessed using concentration-response stimulation with DEANO in 2 µM PE-preconstricted aortic ring in the presence of 300 μM l-NAME to exclude possible endothelial-dependent NO effects **(C)**. Statistical analysis was preformed using two-way ANOVA **(A,C)** or student *t*-test **(B)**. Only the overall significance (bottom left) of ANOVA tests is listed. ns, *p* > 0.05; *, *p* < 0.05; **, *p* < 0.01; ***, *p* < 0.001.

Since *ex vivo* isometric reactivity was minimally affected by age in eNOS-knockout mice, the data is only presented at 6 months of age. Endothelial NO production was induced by concentration-response stimulation with ACh in 2 µM PE-preconstricted aortic rings, confirming the absence of endothelial-dependent relaxations in eNOS knockout mice (Figure 4A). Similarly, the basal production of endothelial NO was assessed as the relative difference in contractile force induced by 2 μM PE in the absence and presence of NO blocker l-NAME (300 µM), confirming impaired basal NO production in eNOS knockout mice (Figure 4B). Concentration-response stimulation with the exogenous NO donor DEANO in 2 µM PE-preconstricted aortic rings (pretreated with l-NAME to exclude effects from endogenously produced NO), showed increased sensitivity to endothelial-independent vasorelaxation, expressed as a significantly decreased IC_50_ value in eNOS knockout mice (8.24 ± 0.09 log(M), compared to −7.77 ± 0.10 log(M) in C57Bl/6 mice, **) (Figure 4C). Hence, eNOS aortic segments were more sensitive to NO.

### eNOS Knockout Mice Display Altered VGCC Function

**FIGURE 5:**
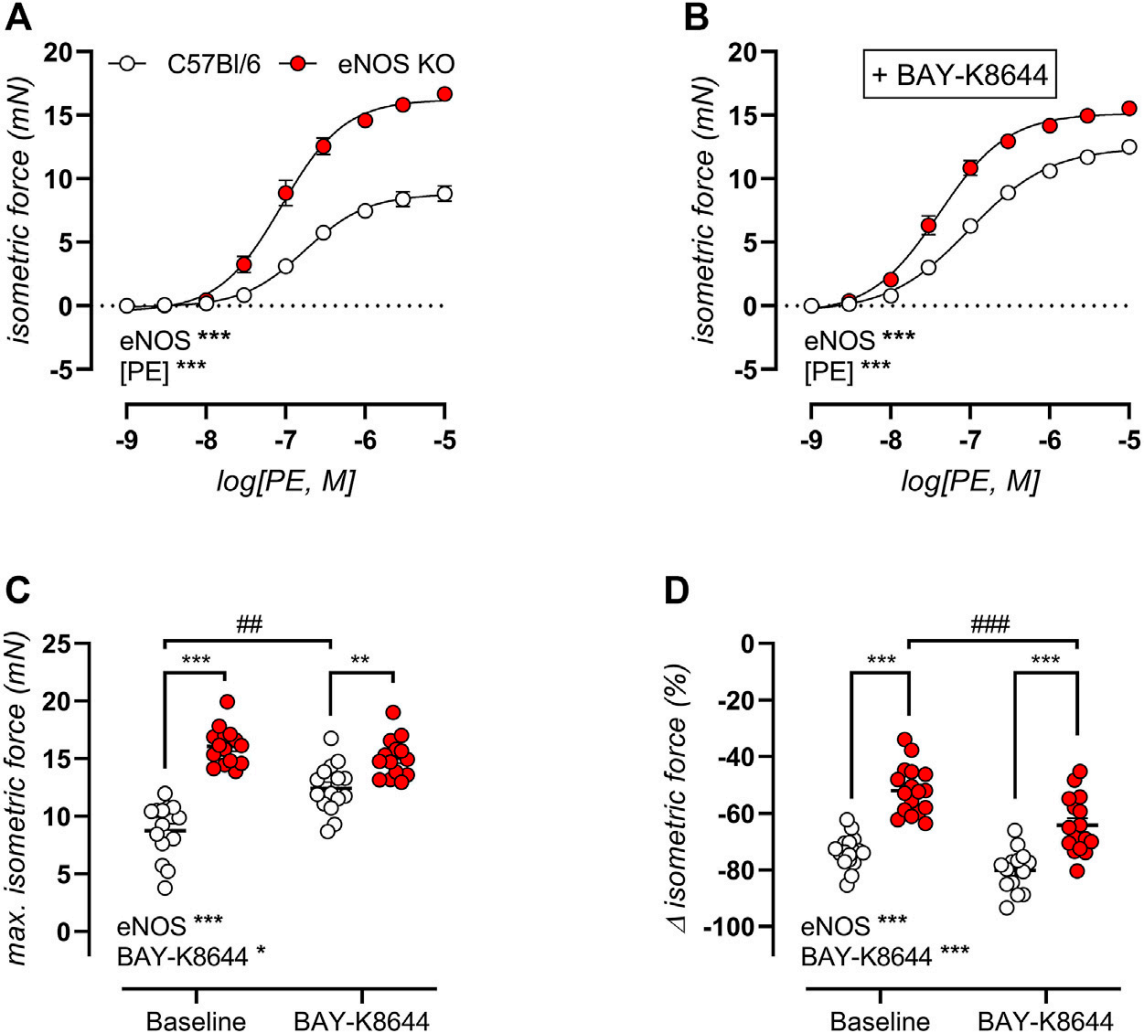
Altered VGCC function in eNOS knockout mice. VGCC function was studied in 6-month old eNOS knockout (*n* = 17) and C57Bl/6 control (*n* = 17) mice, using concentration-response PE-induced isometric contractions in the absence **(A)** and presence **(B)** of VGCC agonist BAY-K8644 (30 nM). The maximal effect of the concentration-response curves in **(A)** and **(B)** was calculated using a non-linear 4-parameter equation **(C)**. VGCC were subsequently inhibited by 35 µM diltiazem to assess the VGCC contribution to PE-induced isometric contractions **(D)**. Statistical analysis was preformed using two-way ANOVA. Overall significance (bottom left) and post-hoc significance (in graph) are listed. No post-hoc significance is listed in **(A,B)**. Overall significance and post-hoc test for genotype: *, *p* < 0.05; **, *p* < 0.01; ***, *p* < 0.001. Post-hoc test for BAY-K8644: ^##^, *p* < 0.01; ^###^, *p* < 0.001.

Concentration-response curves for PE-induced isometric contractions were studied in aortic rings of 6-month old eNOS knockout and C57Bl/6 control mice in the absence (Figure 5A) and presence (Figure 5B) of the VGCC agonist BAY-K8644 (30 nM) to assess VGCC function. Both in the absence and presence of BAY-K8644, PE-induced isometric contractions were significantly higher in eNOS knockout mice. Furthermore, in C57Bl/6 mice, but not in eNOS knockout mice, addition of BAY-K8644 strongly increased maximal PE-induced contractions. This is further illustrated in Figure 5C, which displays the maximal contraction by PE in the absence and presence of BAY-K8644. Next, VGCC were inhibited by 35 µM diltiazem in maximally contracted aortic rings in the absence and presence of BAY-K8644. This revealed that the VGCC contribution to PE-induced isometric contractions was significantly reduced in eNOS knockout mice (in both conditions), and that BAY-K8644 increased this contribution to a larger extent in eNOS knockout mice compared to C57Bl/6 controls (Figure 5D). The latter was unexpected, since maximal PE-induced isometric conditions were not increased by BAY-K8644 stimulation in eNOS knockout mice. Remarkably, we could thus demonstrate the contribution of VGCC to PE-induced isometric contractions in aortic rings of eNOS knockout mice was reduced, whereas the contribution of VGCC to PE-induced aortic stiffening (Figure 3B) was increased.

### eNOS Knockout Mice Display Altered Aortic Basal Tonus

**FIGURE 6:**
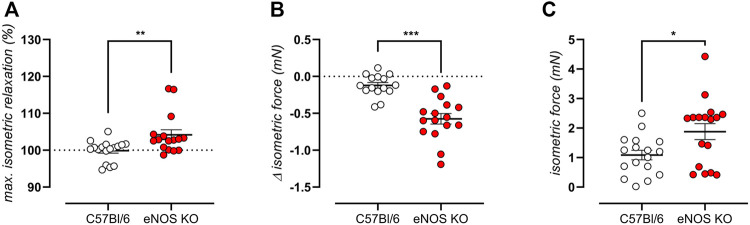
eNOS knockout mice display high VSMC cytoplasmic calcium. VMSC intracellular calcium was studied in 6-month old eNOS knockout (*n* = 16) and C57Bl/6 control (*n* = 15) mice. Increased baseline contractile tone was shown as a >100% maximal relaxation (calculated from the concentration-response curves using a non-linear 4-parameter equation) upon addition exogenous NO donor DEANO **(A)**. Increase cytoplasmic calcium load was demonstrated as a decrease in contractile force when normal Krebs Ringer solution was replaced with a solution lacking extracellular calcium (0Ca) **(B)**. A baseline depolarization was shown as active isometric contraction upon addition of 30 nM BAY-K8644 in Krebs Ringer solutions (since these channels are voltage-dependent, contraction is only initiated in case of depolarization) **(C)**. Statistical analysis was preformed using student *t*-test **(A,B)** or Mann Whitney test **(C)**. *, *p* < 0.05; **, *p* < 0.01; ***, *p* < 0.001.

As previously stated, an age-independent baseline elevated aortic stiffness was shown by biomechanical testing of aortic rings from eNOS knockout mice (Figure 3C). Isometric contractility data confirmed this finding, by showing a significantly >100% relaxation after administration of the exogenous NO donor DEANO (Figure 6A), by showing a significant decrease in contractile force upon removal of extracellular calcium from baseline Krebs-Ringer solution (Figure 6B), and by demonstrating increased isometric contractions upon VGCC stimulation using 30 nM BAY-K8644 in baseline Krebs-Ringer conditions (Figure 6C), all of which were significantly different from C57Bl6 control mice. Taken together, these data suggest increased basal cytoplasmic contractile calcium load, probably due to depolarization of eNOS knockout aortic VSMC.

### eNOS Knockout Mice Display Altered SR-Mediated Transient Contractions

**FIGURE 7:**
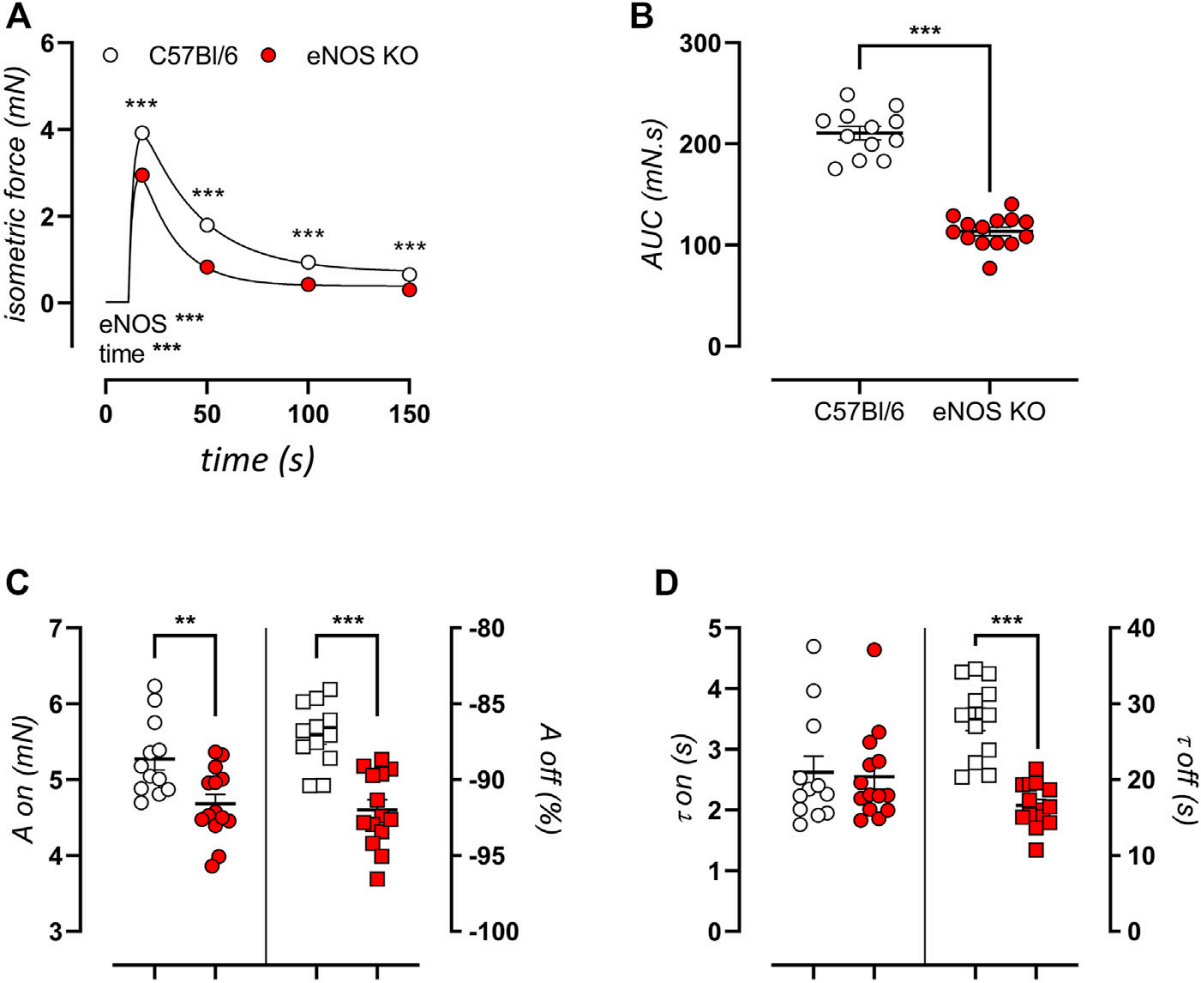
eNOS knockout mice display smaller SR-medicated isometric contractions. Transient SR-mediated contractions were studied in 6-month old eNOS knockout (*n* = 14) and C57Bl/6 control (*n* = 12) mice by stimulation with 2 μM PE in a calcium-free extracellular environment (0Ca Krebs). Presented results are the average of two aortic segments per mouse studied in parallel. A tracing of the contraction **(A)** and calculated area under the curve **(B)** are shown. Bi-exponential fitting of the upward (on) and downward (off) phases of the contraction was used to calculate the amplitude (A) **(C)** and time constant (τ) **(D)** of both phases. The amplitude of the downward phase (A_off_) was calculated as the relative relaxation as compared to the amplitude of the upward phase (A_on_). Statistical analysis was preformed using two-way ANOVA **(A)** or student *t*-test **(B–D)**. Overall significance (bottom left) and post-hoc significance (in graph) are listed. **, *p* < 0.01; ***, *p* < 0.001.

Next, sarcoplasmic reticulum (SR)-mediated contractions were elicited by 2 μM PE stimulation in the absence of extracellular calcium. The resulting transient isometric contraction is attributed to the release of contractile calcium from intracellular stores. Tracings of these contractions are shown in Figure 7A for eNOS knockout mice and C57Bl/6 control mice of 6 months. Contractions were significantly decreased in eNOS knockout mice, which manifested in a decreased area under the curve (Figure 7B). Bi-exponential fitting of the upward (contraction, “on”) and downward (relaxation, “off”) phases of this contraction was used to assess the amplitude (Figure 7C) and time constant (Figure 7D) of each phase. This approach revealed that the attenuated SR-mediated isometric contractions were largely due to a smaller amplitude of the contraction phase and a faster relaxation phase (smaller value of τ_off_) leading to an increased relative amplitude of the relaxation phase.

**FIGURE 8:**
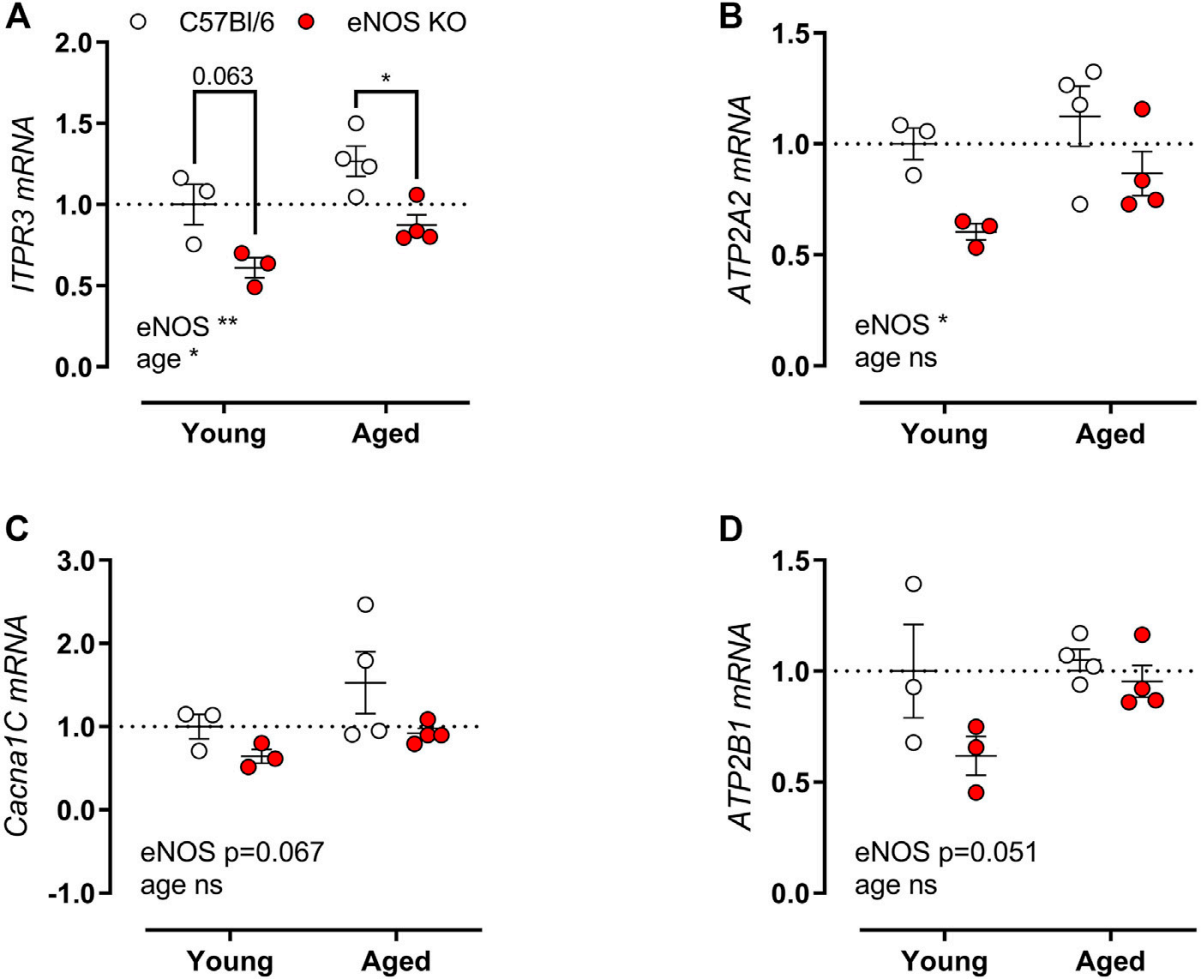
Altered calcium channel gene expression in eNOS knockout mice. Gene expression of ITPR3 **(A)**, Cacna1C **(B)**, ATP2A **(C)**, and ATP2B1 **(D)** was assessed using qPCR on aortic tissue lysates of young (4 months, *n* = 3) and aged (13 months, *n* = 4) eNOS knockout and young (4 months, *n* = 3) and aged (14 months, *n* = 4) C57Bl/6 control mice. mRNA expression was expressed as a fold-change compared to young C57Bl/6 control values. Statistical analysis was preformed using two-way ANOVA. Overall significance (bottom) and post-hoc significance (in graph) are listed. ns, *p* > 0.05, *, *p* < 0.05, **, *p* < 0.01; ***, *p* < 0.001.

Because of the pronounced difference in SR-mediated isometric contractions in eNOS knockout versus C57Bl6 mice, gene expression of the associated calcium channels/pumps was ascertained in aortic tissue from young (4 months) and aged (13 months) eNOS knockout versus age-matched C57Bl/6 control mice. The main calcium channels/pumps that determine the SR-mediated contractions are the sarcoplasmic/endoplasmic reticulum calcium ATPase (SERCA), VGCC, inositol 1,4,5-trisphosphate receptor (ITPR), and plasma membrane calcium ATPase (PMCA). qPCR analysis revealed a marked reduction in ITPR3 (Figure 8A) and ATP2A2 (encoding SERCA) (Figure 8B) mRNA expression in eNOS knockout mice. A further non-significant trend towards decreased Cacna1C (encoding VGCC) (*p* = 0.067) (Figure 8C) and ATP2B1 (encoding PMCA) (*p* = 0.051) (Figure 8D) mRNA levels was shown. A significant age-dependent effect was only observed for the expression of ITPR3, which was increased with age, independent of eNOS genotype.

### eNOS Knockout Mice Display Preserved SOCE-Mediated Contractions

**FIGURE 9:**
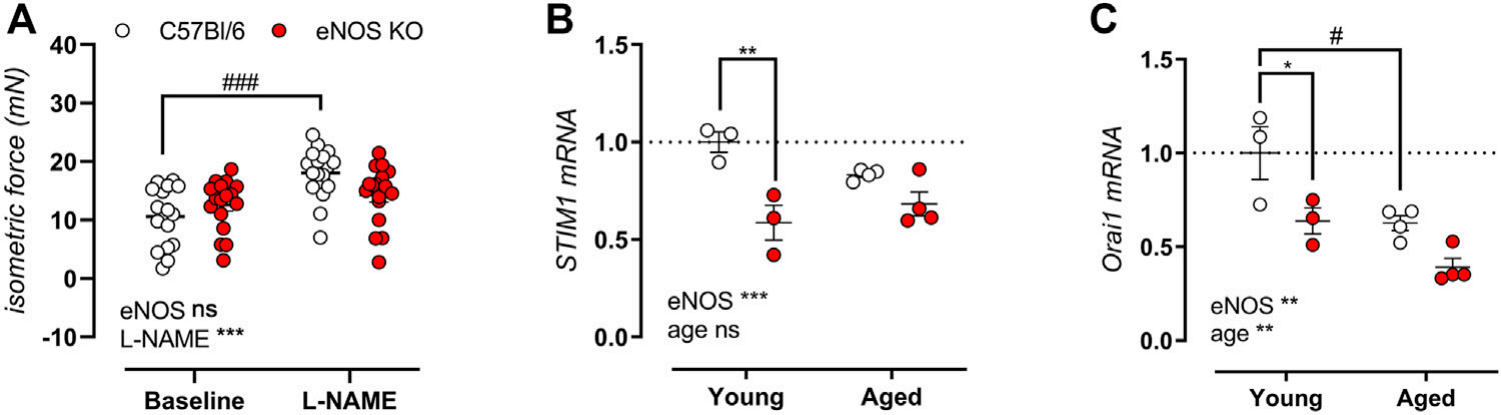
Preserved SOCE-mediated contractions in eNOS knockout mice despite reduced gene expression of STIM1 and Orai1. SOCE-mediated contractions were studied in 6-month old eNOS knockout (*n* = 14) and C57Bl/6 control (*n* = 12) in the absence and presence of 300 μM l-NAME to inhibit basal NO production by re-addition of extracellular after emptying of the SR calcium stores **(A)**. Gene expression of STIM1 **(B)** and Orai1 **(C)** was assessed using qPCR on aortic tissue lysates of young (4 months, *n* = 3) and aged (13 months, *n* = 4) eNOS knockout and young (4 months, *n* = 3) and aged (14 months, *n* = 4) C57Bl/6 control mice. mRNA expression was expressed as a fold-change compared to young C57Bl/6 control values. Statistical analysis was preformed using two-way ANOVA. Overall significance (bottom) and post-hoc significance (in graph) are listed. ns, *p* > 0.05, *, *p* < 0.05, **, *p* < 0.01; ***, *p* < 0.001. ###, *p* < 0.001 for post-host testing of the effect of l-NAME in **(A)**.

After emptying of the SR contractile calcium stores by induction of VSMC contraction in the absence of extracellular calcium, store-operated calcium entry (SOCE)-mediated contractions were elicited by re-addition of extracellular calcium. As shown in Figure 9A, isometric SOCE contractile force is shown in the presence and absence of NOS blocker l-NAME, indicating no significant difference in SOCE-mediated isometric contractions. Note that in C57Bl/6 control mice, SOCE-mediated contraction were significantly increased upon inhibition of basal NO production, whereas no increase in SOCE-mediated contractions was observed in eNOS knockout mice. Interestingly, gene expression analysis revealed a pronounced reduction in stromal interaction molecule 1 (STIM1) (Figure 9B) and calcium release-activated calcium modulator 1 (Orai1) (Figure 9C), the main players in SOCE. With age, the expression of STIM1 and Orai1 further decreased, especially in C57Bl/6 mice.

### Baseline Aortic Stiffness Is Dependent on eNOS, Age, and Cyclic Stretch Amplitude

**FIGURE 10:**
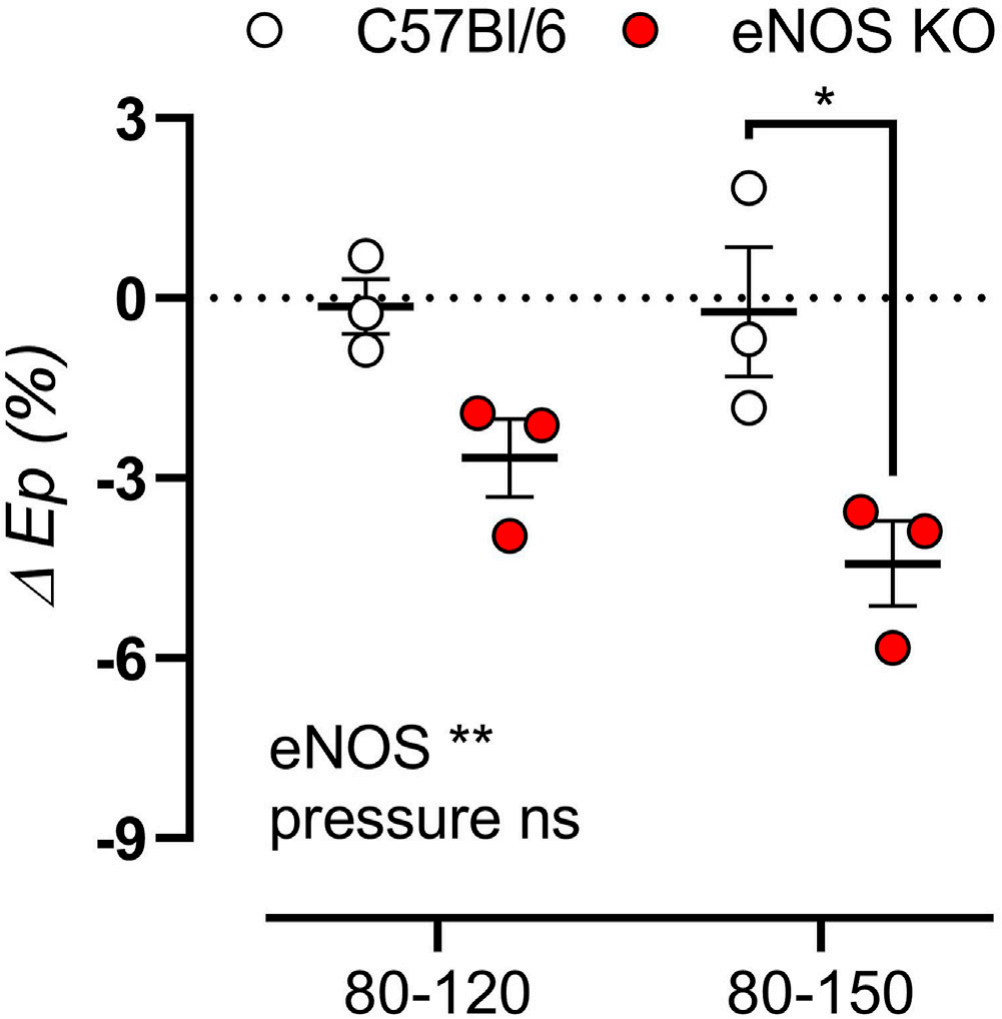
Aortic stiffness in eNOS knockout mice decreases after cyclic stretch exposure. Aortic rings of eNOS knockout (*n* = 3) and C57Bl/6 control (*n* = 3) mice were exposed to 1 h cyclic stretch at calculated 80–120 mmHg or 80–150 mmHg pressures. The relative difference in E_p_ after exposure was calculated. Presented results are the average of two aortic segments per mouse studied in parallel. Statistical analysis was preformed using two-way ANOVA. Overall significance (bottom) and post-hoc significance (in graph) are listed. ns, *p* > 0.05, *, *p* < 0.05, **, *p* < 0.01.

To investigate whether aortic stiffness in eNOS knockout mice was dependent on *ex vivo* cyclic stretch exposure, aortic segments of eNOS knockout and C57Bl6 mice were exposed to 1 h cyclic stretch at calculated 80–120 mmHg or 80–150 mmHg pressures to investigate the effect of cyclic stretch on aortic stiffness. Interestingly, the relative difference in E_p_ after cyclic stretch exposure showed a decrease, selectively in eNOS knockout mice, and this effect was more pronounced at high pulse pressure (80–150 mmHg) (Figure 10).

**FIGURE 11:**
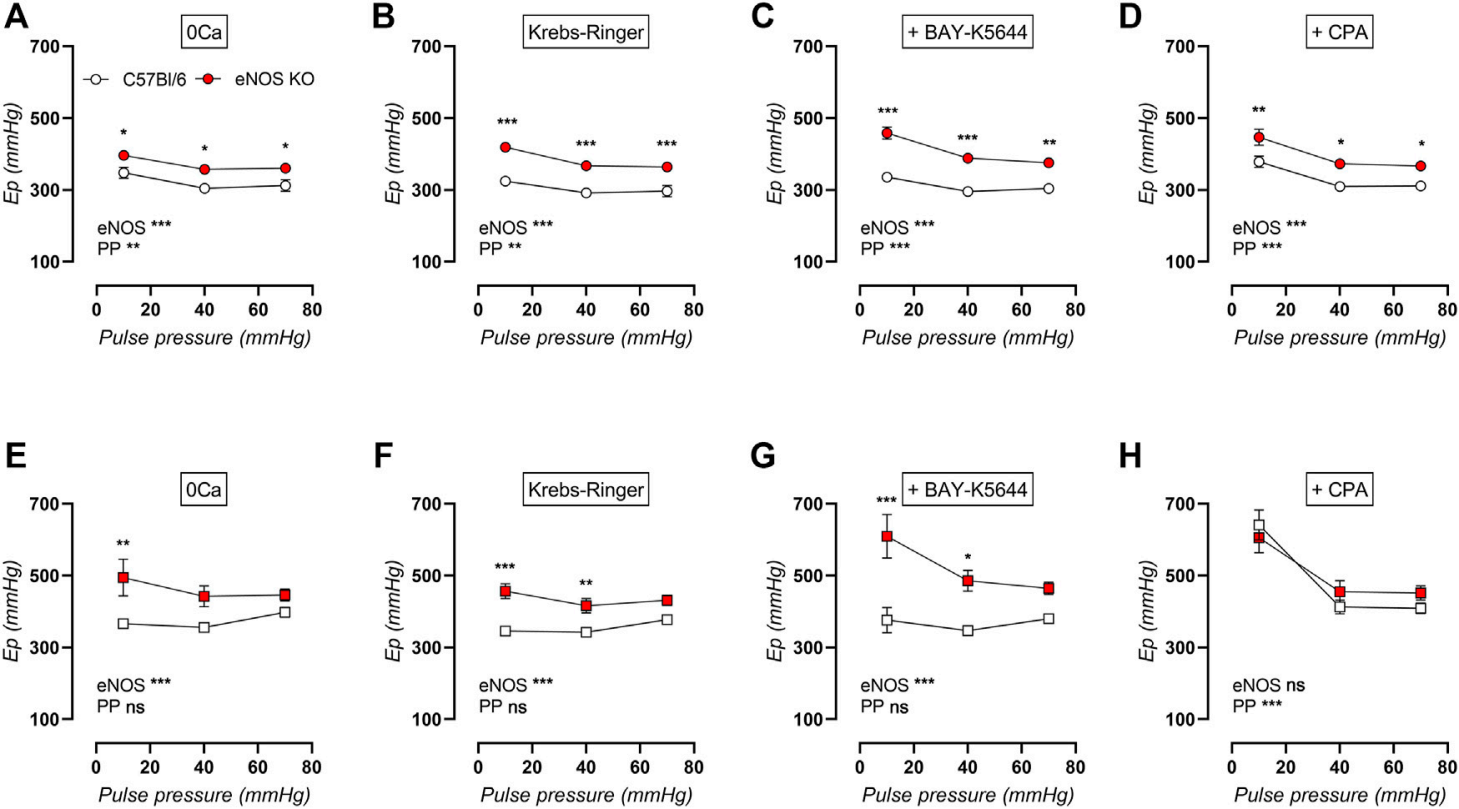
Cyclic stretch-dependency of baseline aortic stiffness is better preserved with age in eNOS knockout mice. Aortic rings of young (4 months, *n* = 6) and aged (13 months, *n* = 4) eNOS knockout and young (4 months, *n* = 6) and aged (14 months, *n* = 4) C57Bl/6 control mice were subsequently exposed to 5 min of cyclic stretch at low (10 mmHg), physiological (40 mmHg), and high (70 mmHg) pulse pressure (PP). Aortic stiffness of young (circles, **(A–D)**) and aged (squares, **(E–H)**) mice was measured in 0Ca Krebs-Ringer solution **(A,E)**, normal Krebs-Ringer solution **(B,F)**, in the presence of 30 nM BAY-K8644 **(C,G)**, and in the presence of 10 µM CPA **(D,H)**. Statistical analysis was preformed using two-way ANOVA. Overall (bottom) and post-hoc (in graph) significance are listed. ns, *p* > 0.05; *, *p* < 0.05; **, *p* < 0.01; ***, *p* < 0.001.

Because of the distinct alterations in VSMC calcium signaling in eNOS knockout mice previously described in this study, it was further investigated whether VSMC calcium channel function was altered by cyclic stretch stimulation. Therefore, measurements of baseline stiffness were performed in young and aged eNOS knockout versus age-matched C57Bl/6 control mice in the absence of extracellular calcium (0Ca, Figures 11A,E), in normal Krebs-Ringer solution (Figures 11B,F), after addition of VGCC agonist BAY-K8644 (Figures 11C,G), and after addition of SERCA pump inhibitor CPA (Figures 11D,H). Furthermore, baseline aortic stiffness was investigated after exposure to various cyclic stretch amplitudes (i.e., 80–90 mmHg, 80–120 mmHg, and 80–150 mmHg) to investigate the cyclic stretch-dependency of aortic stiffening. Overall, baseline aortic stiffness was increased in eNOS knockout mice and in aged versus young mice, and showed a clear cyclic stretch-dependency, with higher E_p_ at low cyclic stretch amplitude. Interestingly, this cyclic stretch-dependency was more pronounced in young mice than in aged mice, in both eNOS knockout and C57Bl/6 control mice. However, cyclic stretch-dependency seemed better preserved with age in aortic rings of eNOS knockout mice, amplifying the difference between eNOS knockout and C57Bl/6 mice at low pulse pressure. This was most pronounced after stimulation of baseline calcium influx through addition of VGCC agonist BAY-K8644 in aortic rings of aged eNOS knockout and C57Bl/6 mice (Figure 11G), indicating that VSMC of aged eNOS knockout mice are more sensitive to calcium influx upon VGCC-activation at low stretch. Contrarily, the increase in aortic stiffness due to eNOS knockout was abolished in aged mice in the presence of SERCA pump inhibitor CPA (Figure 11H), meaning that SERCA pump inhibition differently affected aortic stiffness in aged eNOS knockout and C57Bl/6 mice.

**FIGURE 12:**
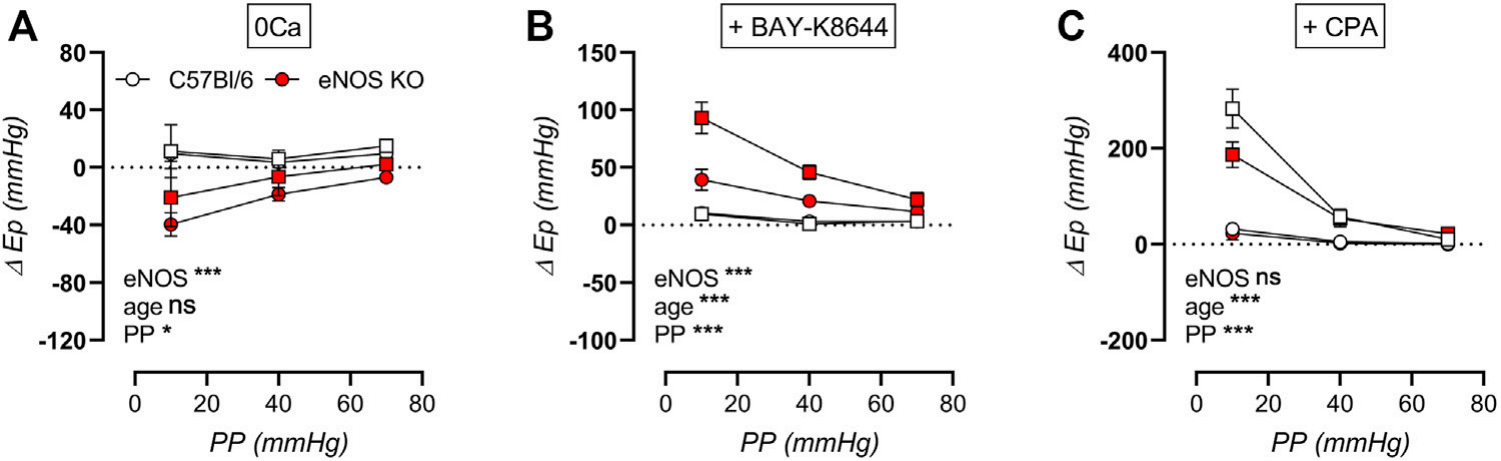
Stretch dependency of VSMC tone and intracellular calcium. Aortic rings of young (4 months, *n* = 6) and aged (13 months, *n* = 4) eNOS knockout and young (4 months, *n* = 6) and aged (14 months, *n* = 4) C57Bl/6 control mice were subsequently exposed to 5 min of cyclic stretch at low (10 mmHg), physiological (40 mmHg), and high (70 mmHg) pulse pressure (PP). Extracellular calcium was removed using 0Ca Krebs Ringer solution to abolish active VSMC tone **(A)**. Aortic rings were stimulated with 30 nM BAY-K8644 **(B)** or 10 µM CPA (in the presence of 300 μM l-NAME) **(C)** in baseline Krebs-Ringer conditions. Statistical analysis was preformed using thee-way ANOVA. Only the overall significance (bottom) is listed. ns, *p* > 0.05; *, *p* < 0.05; ***, *p* < 0.001.

The cyclic stretch dependency of baseline calcium-dependent aortic stiffening due to extracellular calcium removal, VGCC stimulation, and SERCA pump inhibition on aortic stiffening was further investigated, by calculating the difference in E_p_ compared to baseline Krebs-Ringer solution (Figure 12). By removing extracellular calcium, baseline VSMC tone is removed, which resulted in a decrease in aortic stiffness in eNOS knockout mice (and not in C57Bl/6 control mice), which was independent of age but highly dependent on cyclic stretch amplitude (Figure 12A). Similarly, BAY-K8644-induced stimulation of baseline calcium influx via VGCC selectively increased aortic stiffness in eNOS knockout mice in a cyclic stretch-dependent manner, and was most pronounced in aged versus young eNOS knockout mice (Figure 12B). Contrarily, inhibition of calcium re-uptake to the SR selectively increased aortic stiffness in aged and not in young mice, independent of genotype, but again highly dependent on cyclic stretch amplitude (Figure 12C). Taken together, baseline aortic stiffness and, hence, related intracellular baseline calcium levels are highly dependent on cyclic stretch amplitude, both by affecting calcium influx (VGCC) and calcium reuptake (SERCA), although these were diversely affected by eNOS genotype and age.

### Active (Contraction-Dependent) Aortic Stiffness Is Also Dependent on eNOS, Age, and Cyclic Stretch Amplitude

**FIGURE 13:**
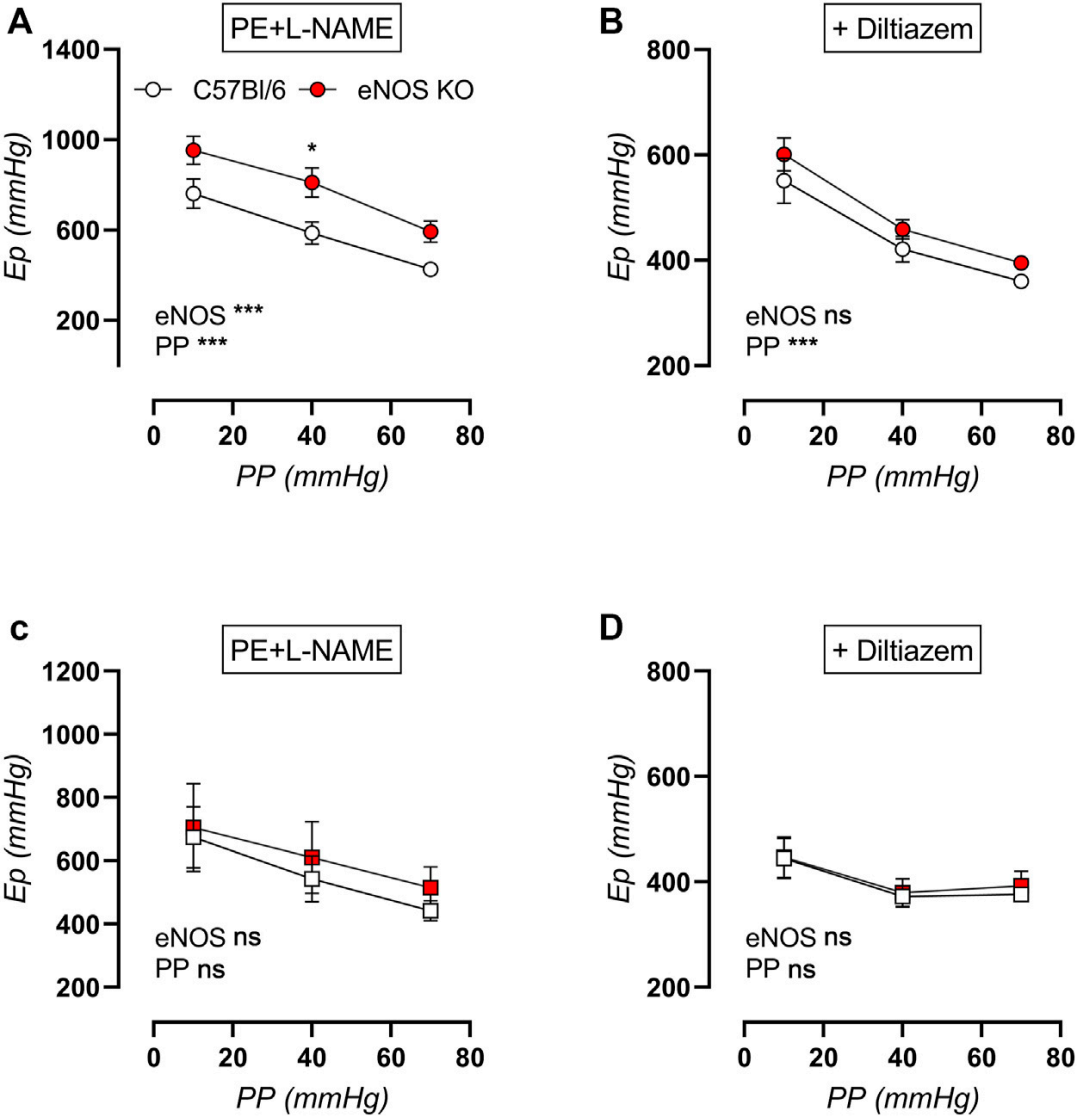
Cyclic stretch-dependency of active aortic stiffness in 2 µM PE-contracted aortic rings. Aortic rings of young (4 months, *n* = 6) and aged (13 months, *n* = 4) eNOS knockout and young (4 months, *n* = 6) and aged (14 months, *n* = 4) C57Bl/6 control mice were subsequently exposed to 5 min of cyclic stretch at low (10 mmHg), physiological (40 mmHg), and high (70 mmHg) pulse pressure (PP). Active aortic stiffness of young (circles, **(A,B)**) and aged (squares, **(C,D)**) mice was measured in 2 µM PE-constricted aortic rings in the presence of 300 μM l-NAME to inhibit basal NO production **(A)**. Subsequently, 35 µM diltiazem was added to inhibit VGCC-dependent aortic stiffening **(B)**. Statistical analysis was preformed using two-way ANOVA. Overall (bottom) and post-hoc (in graph) significance are listed. ns, *p* > 0.05; *, *p* < 0.05; ***, *p* < 0.001.

Active aortic stiffness was investigated in contracted aortic rings after exposure to various cyclic stretch amplitudes (i.e., 80–90 mmHg, 80–120 mmHg, and 80–150 mmHg) to investigate the cyclic stretch-dependency of active aortic stiffness in young and aged eNOS knockout versus C57Bl/6 control mice. Aortic contraction was induced by 2 μM PE (in the presence of 300 μM l-NAME to exclude the difference in basal NO production) (Figures 13A,C), and was subsequently partially relaxed with 35 µM diltiazem to assess the contribution of VGCC (Figures 13B,D). Active aortic contraction increased aortic stiffness compared to baseline values in both genotypes and at both ages. Similar to the baseline aortic stiffness, active aortic stiffness showed a clear cyclic stretch-dependency, with higher E_p_ at low cyclic stretch amplitude, and cyclic stretch-dependency was greater in young mice compared to aged mice. Increased aortic stiffness in eNOS knockout mice was most pronounced in young mice, and was attenuated and no longer significantly increased in aged eNOS knockout mice. Interestingly, addition of 35 µM diltiazem greatly attenuated the difference in active aortic stiffness in young eNOS knockout mice (Figure 13B) and completely reduced active aortic stiffness to C57Bl/6 values in aged eNOS knockout mice (Figure 13D).

**FIGURE 14:**
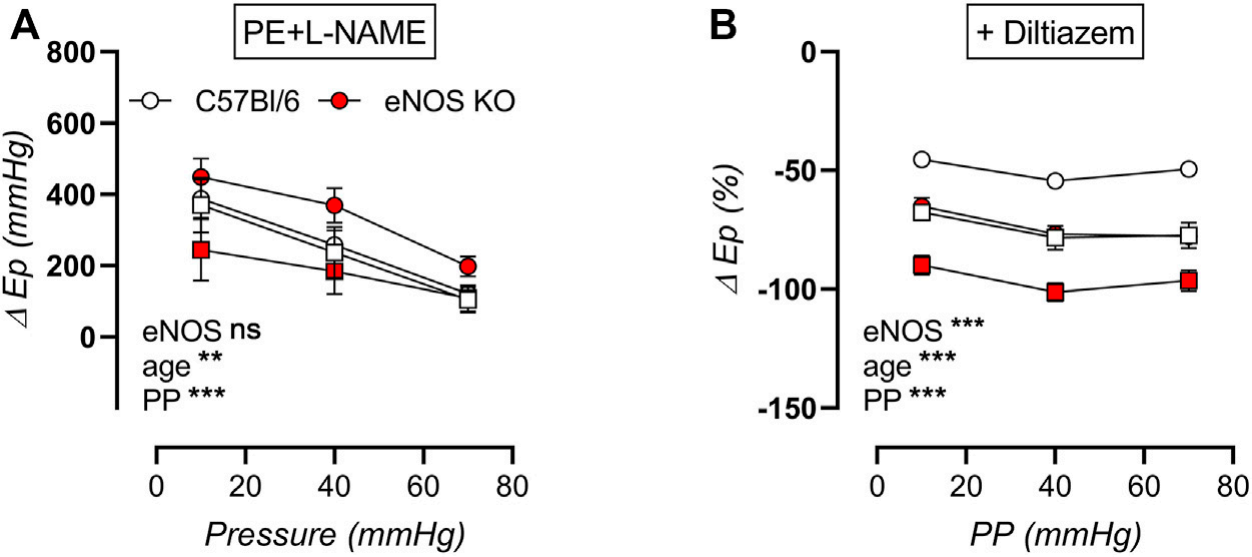
Stretch dependency of contraction-dependent aortic stiffening. Aortic rings of young (4 months, *n* = 6) and aged (13 months, *n* = 4) eNOS knockout and young (4 months, *n* = 6) and aged (14 months, *n* = 4) C57Bl/6 control mice were subsequently exposed to 5 min of cyclic stretch at low (10 mmHg), physiological (40 mmHg), and high (70 mmHg) pulse pressure (PP). Contraction-dependent aortic stiffening by 2 μM PE was plotted **(A)**, and the relative inhibition of 2 µM PE-induced aortic stiffening by addition of 35 µM diltiazem was assessed as measure of VGCC contribution **(B)**. Statistical analysis was preformed using three-way ANOVA. Only the overall significance (bottom) is listed. ns, *p* > 0.05, **, *p* < 0.01; ***, *p* < 0.001.

The increase in aortic stiffness due to stimulation of aortic rings with 2 μM PE (in the presence of NOS blocker l-NAME) was plotted in Figure 14A, showing strong cyclic stretch dependency in all groups, with reduced contraction-dependent aortic stiffening at high pulse pressure. Active aortic stiffening was smallest in aged eNOS knockout mice, and largest in young eNOS knockout mice, whereas age did not seem to affect PE-induced aortic stiffening of C57Bl/6 control mice. The contribution of VGCC to PE-induced active aortic stiffening was assessed via inhibition with diltiazem (Figure 14B), showing that this contribution was increased in eNOS knockout mice. Furthermore, it was augmented with age, independent of genotype, reaching a maximal 80–100% inhibition of aortic stiffening in aged eNOS knockout mice. Furthermore, the VGCC contribution was sensitive to cyclic stretch amplitude in all groups, with a largest contribution at physiological pulse pressure, which was slightly attenuated at both low and high cyclic stretch amplitude. Taken together, it can be concluded that the cyclic stretch dependency of active aortic stiffening/de-stiffening was similar at each age and for both genotypes.

## Discussion

In the present study, we describe the alterations in aortic physiology and biomechanics, underlying the pronounced CV disease phenotype in eNOS knockout mice. Although previously described by our research group (Leloup et al., 2020), the present study now reports a longitudinal characterization of eNOS knockout and age-matched C57Bl/6 control mice at 2, 4, 6, 9, and 12 months of age, thereby investigating whether eNOS knockout mice display progression of aortic disease and its disease mechanisms. Furthermore, a thorough physiological investigation is presented of how modulation of VSMC calcium signaling affects isometric contraction and aortic biomechanics in eNOS knockout mice, with an emphasis on how these are affected by age and cyclic stretch-mechanotransduction.

### eNOS Knockout Mice Display Limited Age-Related Disease Progression

CV disease in eNOS knockout mice and age-matched C57Bl/6 control mice was studied from 2 to 12 months of age. Over this period, C57Bl/6 mice displayed significant CV aging, including progressively increasing aortic stiffness (aPWV and E_p_) without a change in peripheral blood pressure. Furthermore, physiological investigation revealed heightened α_1_-adrenergic contraction-dependent aortic stiffening and a concomitantly increased contribution of VGCC to contraction-dependent aortic stiffening with ageing. Although these parameters were all increased in eNOS knockout mice from a young age onward, further age-dependent effects were reduced or absent, or displayed a similar age-dependent slope as observed in C57Bl/6 control mice. This indicates attenuated age-dependent disease progression in eNOS knockout mice. To our best knowledge, this is the first study that describes the lack of age-dependent progression of arterial disease parameters in eNOS knockout mice. In the heart, a similar lack of age-dependent disease development has been described in eNOS knockout versus C57Bl/6 mice when studying the development of cardiac hypertrophy (assessed as left-ventricular wall and interventricular septum thickening) from 4 to 12 months of age (Li et al., 2004).

### Calcium Influx via VGCC Inversely Affects Isometric Contraction and Aortic Stiffening in eNOS Knockout Mice

VGCC represent the foremost calcium entry pathway in VSMC, illustrated by the pronounced phenotype of SMC-specific Cacna1C knockout mice (i.e., disestablished myogenic tone and severe hypotension) (Moosmang et al., 2003). In the present study, we observed that, in isometric conditions, relaxation of PE-preconstricted aortic rings with the VGCC blocker diltiazem was about ∼50% in eNOS knockout mice, versus ∼75% inhibition in C57Bl/6 mice (at 6 months of age). The reduced contribution of VGCC to PE-induced isometric contraction in eNOS knockout mice may coincide with the non-significant trend towards decreased gene expression of Cacna1C in eNOS knockout mice (*p* = 0.067). Contrarily, relaxations of PE-preconstricted aortic rings with VGCC blocker diltiazem induced ∼75% inhibition of active contraction-dependent aortic stiffening in eNOS knockout mice, versus ∼55% inhibition in C57Bl/6 control mice (at 6 months of age), indicating an inverse role for VGCC in the contribution to aortic stiffening and aortic contraction in eNOS knockout mice. This could be explained by the fact that calcium entry upon VGCC activation affects aortic stiffness in more ways than only by inducing contraction. Aside from activating VSMC contraction, intracellular calcium has a crucial role in regulating VSMC elasticity and adhesion through regulation of the extracellular matrix (ECM)-integrin-cytoskeletal axis (Zhu et al., 2019), by promoting cytoskeletal protein and integrin production (Zhu et al., 2018) and by affecting calcium-sensitive focal adhesion proteins (Hendey and Maxfield, 1993; Sugiyama et al., 2000; Mikami et al., 2017; Zhu et al., 2019). Interestingly, Huang et al. described increased cellular stiffness and adhesion of cultured primary VSMC upon KCl-induced calcium entry, independent of its effects on VSMC contraction (Huang et al., 2018). Based on these findings, the increased contribution of VGCC to aortic stiffening in eNOS knockout mice, despite its reduced contribution to isometric contractions, might result from increased sensitivity to focal adhesion signaling upon VGCC-dependent calcium entry. Additionally, increased calcium-dependent focal adhesion in eNOS knockout mice could also contribute to the increased baseline (non-contracted) isobaric stiffness observed in these animals. Although the role of altered VSMC adhesion in eNOS knockout was not investigated in the present study, similar effects have been described in other cardiovascular disease or aging models (Zhu et al., 2012; Sehgel et al., 2015).

### Altered Intracellular Calcium Handling in eNOS Knockout Mice

The present study confirmed previous findings that eNOS knockout mice display increased basal cytosolic calcium, VSMC depolarization, and active aortic tone (Leloup et al., 2020). In aortic rings of eNOS knockout mice, basal NO production is absent. Because NO hyperpolarizes VSMC through cyclic guanosine monophosphate (cGMP)-dependent activation of ATP-sensitive potassium (K_ATP_) channels, VSMC depolarization and increased basal cytoplasmic calcium are an expected consequence of genetic deletion of eNOS. Furthermore, in healthy arteries, low VSMC cytosolic calcium levels are maintained by calcium export to the extracellular space through PMCA pump activity and intracellular calcium storage in the SR through SERCA pump activity. In the present study, we report significantly reduced ATP2A2 (encoding SERCA) gene expression and a non-significant trend towards decreased ATP2B1 (encoding PMCA) gene expression (*p* = 0.051), further contributing to high cytosolic calcium in eNOS knockout aortic VSMC.

VSMC intracellular calcium handling was also investigated by measurement of SR-mediated phasic contractions. These contractions were induced by 2 μM PE in the absence of extracellular calcium, and therefore correspond to the release of contractile calcium from intracellular stores. As previously shown (Leloup et al., 2020), SR-mediated contractions were reduced in eNOS knockout mice. This was mostly due to a decreased amplitude of the contraction phase, which is dependent on IP_3_-mediated release of contractile calcium from the SR. This could be explained by decreased filling of the SR or by inefficient emptying of the SR upon IP_3_-stimulation. SR filling is mediated by SERCA pump activity (Fransen et al., 2020), and is highly dependent on the basal activity of VGCC, as previously shown (Leloup A. J. et al., 2015). In the present study, we describe decreased mRNA expression of Cacna1C and ATP2A2 in aortic lysates of eNOS knockout mice, which concurs with decreased SR filling. SR emptying, on the other hand, is mediated by ITPR activity and is highly dependent on the calcium concentration gradient over the SR membrane. The present study shows decreased expression of ITPR3 mRNA and increased cytosolic calcium levels in the aorta of eNOS knockout mice. Therefore, the decreased amplitude of the contraction phase of the SR-mediated contractions in eNOS knockout mice might result from both decreased SR filling and/or inefficient SR emptying. Furthermore, the rate of the relaxation phase was increased in eNOS knock out mice and this might correspond to an increased rate of calcium removal from the cytoplasm to the extracellular space. The calcium removal phase is highly dependent on the activity of the PMCA pump (Leloup A. J. et al., 2015). The finding of increased calcium export upon SR-mediated contraction is therefore in apparent conflict with the observation of reduced gene expression of ATP2B1. However, PMCA activity is regulated on many levels (Di Leva et al., 2008), including regulation via splice variation (Strehler and Zacharias, 2001), interaction with calmodulin (Elwess et al., 1997), phosphorylation by PKC (Enyedi et al., 1996) or protein kinase A (PKA) (Guerini et al., 2003), and ATP availability (Di Leva et al., 2008). Gene expression of ATP2B1 may therefore not be representative of its overall activity. Furthermore, calcium export over the plasma membrane can also occur through activity of the Na^+^/Ca^2+^ exchanger. As expression of this protein was not investigated in the present study, it is unclear to which extent it contributes to the calcium export in VSMC of eNOS knockout mice. To further investigate eNOS intracellular calcium handling to differentiate between both options, it could be interesting to measure calcium more directly.

### Cyclic Stretch Regulation of VSMC Intracellular Calcium

The present study further showed that high basal aortic stiffness and isometric tonus likely correspond to increased VSMC calcium levels. We previously reported that in the healthy mouse aorta, removal of extracellular calcium did not decrease basal force at physiological static stretch, but significantly decreased basal force after exposure to high static stretch (De Moudt et al., 2017). Basal aortic stiffness was significantly higher in eNOS knockout than C57Bl/6 mice. However, the effects of calcium removal were larger in eNOS knock out mice and were stretch-dependent with increased basal cytosolic calcium after low amplitude cyclic stretch exposure, selectively in the eNOS knockout mouse aorta. Hence, static and cyclic stretch affect VSMC contractile calcium differently. Cyclic stretch-dependent regulation of VSMC cytosolic calcium was confirmed by several studies (Davis et al., 1992; Mohanty and Li, 2002; Lindsey et al., 2008; Gilbert et al., 2014).

Although VGCC represent the major calcium entry pathway in VSMC, other mechanism of calcium influx affect vascular reactivity. This includes store-operated calcium entry (SOCE), mediated by an interaction between STIM1, a luminal calcium sensor located on the SR, and Orai1, a calcium channel expressed on the plasma membrane (Wang et al., 2017). Orai1 is activated by STIM1 upon SR calcium store depletion. In VSMC, SOCE pathways are involved in the regulation of vascular contractility, proliferation, and migration (Avila-Medina et al., 2018), and are essential in preserving VSMC SR calcium homeostasis (Manjarres et al., 2010). Alterations in SOCE have been described in vascular disease, such as diabetic vasculopathy (Yang et al., 2020), vascular calcification (Ma et al., 2019), hypoxia (Wang et al., 2017), essential hypertension (Giachini et al., 2009), atherosclerosis (Van Assche et al., 2007), and restenosis (Zhang et al., 2011). In the present study, decreased gene expression of Orai1 and STIM1 were observed in aortic tissue lysates of eNOS knockout mice, although no functional effect of the decreased expression was observed on SOCE-mediated isometric contractions. The expression of Orai1 and STIM1 was also significantly decreased with age, independent of eNOS genotype. A decrease in aortic Orai1 expression with age was confirmed by Yang et al. (2015) who further noted that this reduction was specific to conduit arteries. Interestingly the reduced Orai1 and STIM1 expression in aged mice seemed to affect cyclic stretch-sensitive activation SOCE-mediated aortic stiffening in the present study. In VSMC, Orai1- and STIM1-dependent activation of SOCE can be elicited by stimulation with SERCA pump inhibitor CPA (Ng et al., 2010). In the present study, it was shown that CPA-stimulated aortic rings displayed significant aortic stiffening at low cyclic stretch exposure, specifically in aged mice and independent of eNOS genotype (thereby matching the pattern of Orai1 and STIM1 gene expression reduction) and resembling the CPA effects in mice in which SERCA2a was replaced with SERCA2b (Fransen et al., 2020). Our research group previously showed that SOCE in the healthy mouse aorta is insufficient to elicit significant aortic contractions (Fransen et al., 2020). This indicates that in aged arteries, reorganization of the contractile and/or adhesive apparatus of VSMC results in a higher calcium sensitivity, resulting in active aortic stiffening upon SOCE. CPA-induced aortic stiffening was most pronounced at low cyclic stretch and was significantly attenuated with increasing cyclic stretch exposure. Orai1 and STIM1 interact with the non-selective calcium permeable transient receptor potential canonical (TRPC) channels (Lopez et al., 2020), of which the TRPC1 (Ohba et al., 2007; Seth et al., 2009), TRPC3 (Dalrymple et al., 2007), and TRPC6 (Kuwahara et al., 2006; Niizeki et al., 2008) homologues were shown to be mechanosensitive in smooth muscle cells. Furthermore, stretch-dependent stimulation of smooth muscle cells was demonstrated to affect SOCE though regulation of TRPC channels (Dalrymple et al., 2007). Therefore, although not specifically investigated in the present study, mechanosensitive TRPC activity might explain cyclic stretch-dependent activation of SOCE-mediated aortic stiffening in aged aortic rings.

### Cyclic Stretch Regulation of VSMC Contractility

The cyclic pumping of the heart creates numerous mechanical stimuli to which all cells in the vascular wall are exposed, including transmural pressure, circumferential wall tension, shear stress, and axial tension (Ahmed and Warren, 2018). VSMC are highly subjected to cyclic stretch, which lengthens the cells by approximately 10% with each cardiac ejection (Gosling and Budge, 2003; Gao et al., 2014). These stretch signals regulate the activity of several important signaling mediators such as PKC, Akt, and Rho family GTPases, thereby regulating a broad range of functions, including proliferation, migration, and cell survival (Ahmed and Warren, 2018). The present study describes the cyclic stretch-dependent regulation of active stiffening due to VSMC contraction and calcium signaling in young and aged eNOS knockout mice versus age-matched C57Bl/6 control mice. A pronounced decrease in α_1_-adrenoreceptor mediated aortic stiffening with increasing cyclic stretch amplitude was observed in both genotypes. This is consistent with previous findings by our research groups which demonstrated reduced α_1_-adrenoreceptor mediated isometric contractions after both increased static (De Moudt et al., 2017) and cyclic (Leloup et al., 2017) stretch. Aside from calcium-dependent activation of VSMC contractions, Rho kinase-dependent inhibition of myosin light chain phosphatase constitutes an important determinant of VSMC tone (Puetz et al., 2009). Aside from affecting contraction, small GTPases of the Rho family also regulate actin cytoskeleton remodeling and contractile stress-transmission to ECM via actin polymerization (Albinsson et al., 2004). Cyclic stretching in rat VSMC was shown to alter the expression and localization of focal adhesion components paxillin and vinculin (Cunningham et al., 2002; Na et al., 2008), suggesting that cyclic stretch-mechanotransduction results in strengthened focal adhesion complexes.

The present study further demonstrated that the contribution of VGCC to PE-induced aortic stiffening was sensitive to cyclic stretch. Cyclic stretch-dependent regulation of VGCC activity was confirmed by Zhang et al. (2018). We previously demonstrated a higher sensitivity to VGCC-mediated isometric contraction at high preload static stretch (De Moudt et al., 2017), but smaller VGCC-mediated isometric contractions at high amplitude cyclic stretch (Leloup et al., 2017), again underlining the importance of studying vascular physiology in dynamic conditions. After both static and cyclic stretch exposure, however, no change in VGCC-contribution to α_1_-adrenergic isometric contraction was observed. As previously described, calcium entry upon VGCC activation may induce aortic stiffening by calcium-dependent activation of focal adhesions as well as by initiating VSMC contraction, thereby diversely affecting isometric contraction and active aortic stiffening.

### Role of Endothelial Dysfunction in Cardiovascular Disease

The present study demonstrates that eNOS KO mice, as an absolute and genetic model of endothelial dysfunction, display distinct cardiovascular disease characteristics, even at a very young age. This includes aortic stiffening, hypertension, and pronounced alterations in vasoreactivity and biomechanical responses of the *ex vivo* aorta. As described in the introduction of this paper, eNOS-dependent NO production has a multitude of signaling functions, both within and outside of the vascular system, leading to many systemic alterations after genetic deletion of eNOS. Therefore, the authors acknowledge that not all described alterations in the present study can be ascribed to the local lack of function of endothelial NO in the arterial endothelium. Furthermore, since the systemic loss of eNOS is present from embryological development onward, many compensatory cardiovascular changes have been described in eNOS knockout mice (Mashimo and Goyal, 1999; Godecke and Schrader, 2000; Iafrati et al., 2005). Despite the pronounced and early cardiovascular disease phenotype, limited age-dependent progression of cardiovascular disease was observed up to 12 months of age, whereas C57Bl/6 control mice displayed pronounced age-dependent progression of cardiovascular disease in this age range, as confirmed in previous work by our research group (De Moudt et al., 2020). To our best knowledge, we are the first research group to describe the lack of age-dependent progression of arterial aging in eNOS KO mice.

Endothelial dysfunction is a recognized cardiovascular disease hallmark (Tao et al., 2004; Rossman et al., 2018; Majerczak et al., 2019), which is underlined by the effectiveness of NO-associated therapies, e.g., NO-releasing non-steroidal anti-inflammatory drugs (Fiorucci et al., 2001), statins (Laufs and Liao, 1998; Nakata et al., 2007; Zhang et al., 2012), hormone therapy (Kawano et al., 2003; Williams et al., 2004), resveratrol (Schmitt et al., 2010; Crandall et al., 2012), and dietary factors (Dickinson et al., 2009; McCall et al., 2009; Blumenthal et al., 2010). However, other studies have observed that arterial aging can occur independently of endothelial dysfunction (Del Campo et al., 2019; De Moudt et al., 2020). Interestingly, improved NO function was shown to ameliorate CV disease even in animal models where dysfunctional VSMC signaling is the underlying mechanism of arterial aging (Del Campo et al., 2019). Therefore, we consider that the presented insights into the effects of chronic endothelial dysfunction on aortic biomechanical responses and cyclic stretch-dependent regulation of VSMC calcium levels is of great importance to the field of cardiovascular aging research.

### Study Limitations

In the present study, biomechanical properties of eNOS knockout and C57Bl/6 control mice were studied using in-house developed ROTSAC organ chambers (Leloup et al., 2016), which allows for the investigation of aortic stiffness in dynamic conditions at physiological frequency and (calculated) pressures. Although we previously used ROTSAC organ chambers to investigate aortic function after stimulation at various cyclic stretch amplitudes and frequencies, and even though we were able to demonstrate important differences in aortic physiology due to cyclic stretch regulation (Leloup et al., 2017), it is important to note some important limitations of the ROTSAC set-up for complex biomechanical studies. First, aortic rings are mounted and stretched between two wire hooks in a uniaxial direction. Therefore, stretch is not applied homogenously across the aortic tissue as done *in vivo* where the aorta is stretched circumferentially. Using pressure myography, circumferential stretch can be applied *ex vivo* to isolated arterial segments. However, in such a set-up, pressure oscillations are often applied at a subphysiological frequency, which also affects aortic function (Leloup et al., 2017). Furthermore, *in vivo* arteries are subjected to both circumferential and axial stretch. The latter contribution is also not taken into account in ROTSAC organ chambers. Recently, an adapted dynamic pressure myography device was developed, which can apply both circumferential and axial stretch to aortic segments (van der Bruggen et al., 2021). In this novel set-up, it was demonstrated that axial stretch significantly influences arterial stiffness, with increased arterial stiffness at increasing axial stretch. Secondly, it is important to note that in ROTSAC organ chambers, contrary to pressure myography, pressures are not directly measured. Instead pressures are calculated via Laplace law from dynamically applied preloads (Leloup et al., 2016).

Another limitation in the study design of the present study, was that various cyclic stretch amplitudes were applied while maintaining a constant (calculated) “diastolic” pressure of 80 mmHg (i.e., 80–90 mmHg, 80–120 mmHg, and 80–150 mmHg). Although this was a conscious decision, aimed to mimic physiologically increased pulse pressure in conditions of exercise or chronic aortic stiffness, it also means that mean pressure increased from calculated 85–115 mmHg. It is therefore not possible to conclude that all results obtained in the cyclic stretch experiments resulted solely from the difference in cyclic stretch amplitude applied to the aortic segments, since mean distending pressure also affects aortic physiology and biomechanics. It would thus have been interesting to investigate the separate contributions of both factors in the results described in this study. Our research group recently reported that increased pulsatile load in healthy adult mice reduced aortic stiffness and altered VSMC contractile function, independent of mean blood pressure effects (Neutel et al., 2021), suggesting that the observed effects in this study are likely due to cyclic stretch amplitude.

As the present study focused on calcium signaling in VSMC of eNOS knockout aortic rings, it would be very interesting to include a direct measurement of calcium in future studies. Here, only the resultant effects of calcium signaling on isometric contraction and aortic stiffening were assessed. Although it represents an important advantage to assess the functional outcome of signaling pathway alterations as presented in this study, information on the underlying calcium events could strengthen our conclusions greatly, and should therefore be assessed in future research on this topic.

## Conclusion

This study demonstrates a pronounced CV disease phenotype in eNOS knockout mice, with increased peripheral blood pressure, aortic pulse wave velocity, and aberrant VSMC calcium handling, which displayed attenuated disease progression compared to age-matched C57Bl/6 mice. Since increased VSMC tone in eNOS knockout mice could be reduced by *ex vivo* cyclic stretch exposure in an amplitude-dependent manner, we investigated cyclic stretch regulation of aortic stiffness and VSMC calcium signaling, showing that both baseline and active aortic stiffness were highly dependent on cyclic stretch regulation, which was more pronounced in young versus aged mice.

## Data Availability

The raw data supporting the conclusions of this article will be made available by the authors, without undue reservation.
